# Reducing 30-Day Rehospitalization Rates Using a Transition of Care Clinic Model in a Single Medical Center

**DOI:** 10.1155/2017/5132536

**Published:** 2017-08-02

**Authors:** Tamer Hudali, Robert Robinson, Mukul Bhattarai

**Affiliations:** Department of Internal Medicine, Southern Illinois University, Springfield, IL 62704, USA

## Abstract

**Background:**

Rehospitalization for medical patients is common. Multiple interventions of varying complexity have been shown to be effective in achieving that goal with variable results in the literature. For medical patients discharged home, no single intervention implemented alone has been shown to have a sustainable effect in preventing rehospitalization.

**Objective:**

To study the effect of a transition of care clinic model on the 30-day rehospitalization rate in a single medical center.

**Methods:**

Retrospective observational analysis of adult patients discharged home from Memorial Medical Center from September 1, 2014, through December 31, 2014. The primary outcome was to compare hospital readmission rates between patients who followed up with a transition of care (TOC) clinic and those who did not.

**Results:**

The study population included 378 patient discharges. A total of 40 patients (10.6%) were readmitted to the hospital within 30 days of discharge. Patients who attended the TOC clinic had a significantly lower 30-day readmission rates (3.8% versus 11.7%). A Cox regression analysis showed that the TOC clinic attendance had a significant negative predication for readmission (HR 0.186, 95% CI 0.038–0.898, *P* = 0.038).

**Conclusion:**

Adopting a TOC model after discharging medical patients has reduced the readmission rates in our study.

## 1. Introduction

Providing high quality care for patients remains the ultimate goal for all health care providers. One of the main measures to achieve this goal is to decrease preventable adverse events after discharge from the hospital. Forster et al. [1] studied the adverse events caused by medical care after hospitalization discharge. It was approximated that one-quarter of patients develop an adverse event, half of which are preventable. Another study estimated that about 19.6% of Medicare fee-for-service patients are rehospitalized within 30 days of discharge; the authors concluded that this rate is both prevalent and costly among such populations [2]. In 2011, approximately 3.3 million adults were readmitted to the hospital and the associated costs totaled about $41.3 billion [3]. Researchers, hospitals, and policymakers are actively considering refinements to the Hospital Readmission Reduction Program and looking for ways to engage other providers and patients in reducing preventable patient readmissions to the hospital. The shares of hospitals receiving penalties for 30-day readmissions and total fines are both higher in 2015 [4]. It was also suggested that an estimated $12 billion per year is spent on avoidable hospital readmission costs [2, 5]. Based on these statistics, the Medicare Payment Advisory Commission has suggested multiple interventions to prevent avoidable readmissions and is moving to impose financial penalties on hospitals with excess readmissions [5, 6].

Multiple efforts have been initiated to reduce avoidable rehospitalization and to study the contributing factors. Case studies of hospitals with exceptionally low readmission rates highlight that hospitals' environments contribute to their capacity to reduce readmissions. Readmission rates are also influenced by policy environment, local health care markets, membership in integrated systems that offer a continuum of care, and the priorities set by leaders [7]. Parker et al. [8] conducted a systematic review that categorized interventions to reduce readmissions into 4 types: discharge planning protocols, discharge support arrangements, educational interventions, and comprehensive geriatric assessment. The same review showed that none of these discharge arrangements have effects on mortality or the length of stay in the hospital. A more recent systematic analysis reviewed the effect of multiple interventions in reducing rehospitalization within 30 days and demonstrated that no single intervention is effective when implemented by itself [9].

Several studies which focused on specific conditions provide growing evidence that timely outpatient follow-up contributes to reduced rehospitalization [10–18]. A smaller number of studies focused on general hospitalized patients showed conflicting results [9, 19–22]. Despite this fact, multiple transition of care models have implemented timely outpatient follow-up in an attempt to adequately reduce readmissions. One study by Jackson et al. [21] showed that most Medicaid patients do not benefit from early outpatient follow-up. However, the readmission of high risk patients from this population is significantly reduced when they receive adequate follow-up within 1 week [21]. Another study done in a large urban academic center showed that only 49.2% of hospitalized patients get timely primary care provider (PCP) follow-up upon discharge, and patients without timely PCP follow-up have readmission rates 10 times higher than those who do (odds ratio = 9.9, *P* = 0.04) [20]. Other studies showed that outpatient follow-up among general medicine patients does not decrease the rate of readmission [19, 22, 23].

Our study implemented an intervention where, upon discharge, patients scheduled an appointment with a transition of care clinic run by the hospitalist team. We then investigated the effects of this intervention on the 30-day hospital readmission rates of general medical patients.

## 2. Materials and Methods

Institutional review board review for this study was obtained from the Springfield Committee for Research Involving Human Subjects, who determined that it does not meet the criteria for research involving human subjects according to 45 CFR 46.101 and 45 CFR 46.102.

### 2.1. Study Design

This study retrospectively analyzed all patients discharged from the Southern Illinois University general internal medicine teaching service from Memorial Medical Center (September 1, 2014 to December 31, 2014). Memorial Medical Center is a 507-bed, university-affiliated tertiary care center located in Springfield, Illinois, USA.

The electronic medical record system supplied deidentified data on gender, age, diagnosis related group (DRG) code, length of stay, hospital readmission within 30 days, medical comorbidities, amount of hospitalization in the last year, referral to the transition of care clinic, and attendance at the transition of care clinic.

DRG weights are defined by the Centers for Medicare and Medicaid Services on an annual basis and are related to the cost and complexity of inpatient medical care for a specific DRG. DRG weights were assigned to each patient based on the DRG code and served as a marker of the severity of illness. A Charlson comorbidity index (CCI) was calculated for each patient [24].

The transition of care (TOC) clinic is located in the same building as the patients' primary care physicians and has access to all the facility resources. The clinic staff consists of a hospitalist not on wards service that week and a nurse responsible for the management of medical patients. Additionally, two nurses work at the hospital to schedule clinic appointments and to educate patients about the clinic prior to discharge. These nurses also call the patients within two business days to make sure they continue to do well and to confirm the scheduled clinic appointment. Appointments are scheduled for within one week of the time of discharge.

The primary outcome investigated in this study was hospital readmission for any reason within 30 days of discharge. The secondary outcome in the study was the risk of 30-day readmission based on the different primary diagnoses at time of admission.

### 2.2. Statistical Analysis

Attending the TOC clinic was investigated as a predictor of hospital readmission within 30 days. Qualitative variables were compared using Pearson's chi-square or Fisher's exact test and reported as frequency (%). Quantitative variables were compared using the nonparametric Mann–Whitney *U* or Kruskal-Wallis tests and reported as mean ± standard deviation. Rates of readmission-free survival were evaluated by the Kaplan-Meier method. Demographic and clinical variables were included as explanatory variables in a Cox proportional-hazards regression analysis.

Statistical analyses were performed using SPSS version 22 (SPSS Inc., Chicago, IL, USA). Two sided *P* values < 0.05 were considered significant.

## 3. Results

The study population included 378 hospital discharges with an average patient age of 62 years. The majority of the patients in this sample were female (52.4%). Average length of hospital stay was 4.2 days, average DRG weight was 1.36, and average CCI was 5.42. In this sample, 40 patients (10.6%) were readmitted to the same hospital within 30 days of hospital discharge. Patients who were readmitted to the hospital within 30 days of discharge had a higher number of hospital admissions in the last year (1.83 versus 0.891, *P* = 0.002), have more patients admitted with diabetic ketoacidosis (DKA) (5 versus 3, *P* < 0.001), and have a higher Charlson comorbidity index (6.98 versus 5.23, *P* < 0.001) compared to patients who were not readmitted (Table 1).

The rate of readmission-free survival differed between patients who attended the TOC clinic and those who did not (3.8% and 11.7%, resp.). This relationship was explored with Cox regression, which indicated that attending the TOC was a significant negative predictor of hospital readmission (hazard ratio 0.186, 95% CI 0.038–0.898, *P* = 0.038). The risk of readmission was higher in patients admitted with DKA (hazard ration 10.43, 95% CI 3.92–27.77, *P* < 0.001) and chronic obstructive pulmonary disease (COPD) exacerbations (hazard ration 3.65, 95% CI, 1.09–12.23, *P* = 0.036), as shown in Table 2. No other factors were significantly emerged as predictors for hospital readmission (Table 2).

**Figure 1:**
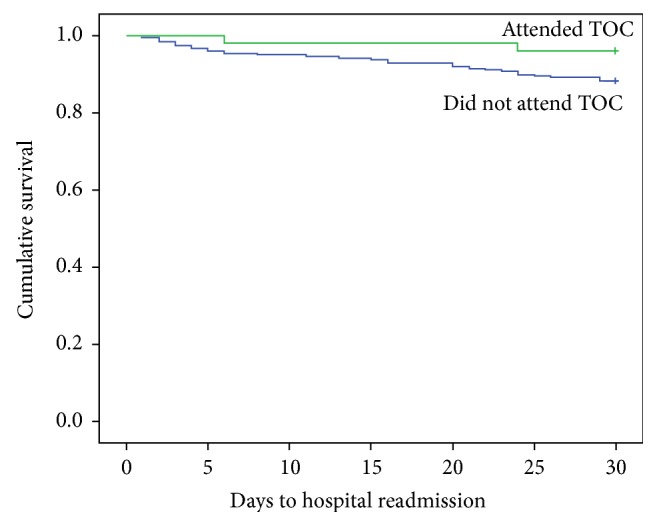
Kaplan-Meier plot comparing 30-day readmission rates between patients who did or did not attend the transition of care clinic.

A Kaplan-Meier plot comparing the readmission-free survival of patients who did or did not attend the TOC is shown in Figure 1.

## 4. Discussion

This study indicates that the utilization of a transition of care clinic after discharge had a potential positive outcome in reducing the readmission rates 30 days after discharge. The reduction of rehospitalization rate observed in our study, from 11.7% to 3.8%, was not only statistically significant but also less than the observed national average for medical patients of 15.9% [25]. This reduction, which was statistically adjusted, is not explained by any other factors studied in the target population. It also indicates that this system, which serves the rural area of southern Illinois, is a peculiar health care system. There are several factors that may have contributed to this reduction, including that early access to medical care helped facilitate patient education and assured the patients' understanding of their complex medical issues; the guarantee of medication reconciliation and access to new prescription upon follow-up; the strengthening of the doctor-patient relationship, especially after the hospital encounter with a strange provider (the hospitalist); reduction of loss of follow-up by providing early appointments; and providing an earlier opportunity to reassess the patient for change in health status.

This intervention goes in line with multiple efforts by hospital administrators and governmental agencies to reduce rehospitalization by proving a high-value care [5]. There is not strong evidence that a single intervention is enough to significantly reduce readmission rates [9], so more complex interventions are required in order to effectively achieve this goal [26]. The heterogeneity of the results found in literature could be a good reflection of the complexity of our healthcare system as well as the dynamic interaction with our complex medical patients. The system of profound knowledge, proposed by the quality improvement pioneer Dr. W. Edwards Deming, supports this assumption. As stated in Deming's book, there are four parts of a system that need to be understood in order to obtain meaningful improvement: appreciation of the system, understanding variation, obtaining a theory of knowledge, and taking psychology into consideration [27].

Our study corroborates previously reported outcomes that timely outpatient visits after discharge, arranged through a transition of care model, reduce readmission rates [11, 16, 20, 28]. Other studies have shown improved rates of readmission among only high risk populations [13, 21] or have demonstrated no clear benefit from the same intervention [19, 22]. These results have been analyzed by Hansen et al. in a systematic review of the interventions used to reduce the 30-day rehospitalization [9]; they concluded that no single intervention is enough to maintain 30-day rehospitalization reduction. Our patient population displays a high CCI that indicates a potentially higher risk of rehospitalization, which may partially explain the observed benefit from using a transition of care model in our institution.

Multiple interventions designed to reduce rehospitalization rates have been previously described. Postdischarge calls are one of the most common interventions that have been widely adopted [9, 26]. The effectiveness of such intervention varies in the literature, again reflecting the complexity of the factors contributing to the rehospitalization process. In our study, the observed effect cannot be assumed to be due to the follow-up calls as all patients, whether they attended the clinic or not, received calls. However, the additive effect of said calls may have contributed to the reduction of the readmission rate. Therefore, we posit that the integration of hospital and outpatient care is key to reducing readmissions. Our hospital's integrated health system contributes to lower admissions and thereby avoids readmissions, through its emphasis on primary and preventive care, community-based education, and enhanced communication and flow of information through easily accessible electronic health records among inpatient and outpatient providers.

Our study has multiple limitations. First, the allocation of patients was not randomized due to lack of appropriate volume of patients and resources. Second, the hospital readmission rate was calculated for a single institution. As patients could have received care from other hospitals in the region, this may not reflect the actual rehospitalization rate. Third, the trial was not blinded, although that is unlikely to affect the results because the outcome measures were objective and extracted from the healthcare records database. Fourth, while our study cannot be safely generalized and applied to other settings, it indicates that a better understanding of current local healthcare systems, identification of local patient characteristics and medical needs, and the proper allocation of resources in the community could help structure appropriate interventions to decrease rehospitalization rates. Fifth, the patients who attend the TOC clinic are the same patients who are likely to be more compliant in their postdischarge care, which also could have a beneficial impact on the readmission risk. Finally, our study was retrospective and observational in nature and thus we cannot assume a causal relationship.

## 5. Conclusion

A smooth transition from the inpatient to the outpatient world constitutes a favorable model of care. Our study demonstrates that adopting a transition of care clinic reduced the readmission rates of our peculiar population. Further studies are warranted to assess the patient population characteristics that benefit from a transition of care clinic model as a method to reduce rehospitalization.

## Figures and Tables

**Table 1:** Patient characteristics.

	Not readmitted *N* = 338	Readmitted *N* = 40	
Age in years (SD)	61.19 (17.72)	66.25 (16.53)	*P* = 0.062
Female gender (%)	175 (52%)	23 (58%)	*P* = 0.493
Length of stay (SD)	4.04 (3.99)	5.58 (5.37)	*P* = 0.053
Hospital admissions in last year (SD)	0.891 (1.48)	1.83 (2.23)	*P* = 0.002
DRG weight (SD)	1.35 (0.85)	1.49 (0.85)	*P* = 0.136
Charlson comorbidity index (SD)	5.23 (3.05)	6.98 (2.90)	*P* < 0.001
Attended TOC clinic (%)	50 (15%)	2 (5%)	*P* = 0.089
Principal diagnosis (ICD-9 code)			
Septicemia NOS (038.9)	36 (11%)	5 (12%)	*P* = 0.722
Cerebral artery occlusion NOS with infarction (434.91)	16 (5%)	1 (2%)	*P* = 0.519
Pneumonia, organism NOS (486)	14 (4%)	2 (5%)	*P* = 0.799
Obstructive chronic bronchitis with acute exacerbation (491.21)	10 (3%)	2 (5%)	*P* = 0.068
Acute and chronic respiratory failure (518.84)	9 (3%)	1 (2.5%)	*P* = 0.952
Acute kidney failure NOS (584.9)	8 (2%)	1 (2.5%)	*P* = 0.958
Subendocardial infarct, initial (410.71)	8 (2%)	0 (0%)	*P* = 0.325
DM type 1 with ketoacidosis, uncontrolled (250.13)	3 (1%)	5 (12%)	*P* < 0.001
Urinary tract infection, NOS (599.0)	7 (2%)	0 (0%)	*P* = 0.358
*E. coli* septicemia (038.42)	6 (2%)	1 (2.5%)	*P* = 0.748

**Table 2:** Cox proportional-hazards regression analysis of risk factors for hospital readmission.

Variable	Regression coefficient	Standard error	Wald	*P* value	Hazard ratio (95% CI)
Age	0.006	0.014	0.157	0.692	1.01 (0.98–1.03)
Gender	0.365	0.335	1.187	0.276	1.44 (0.75–2.78)
Length of stay	0.041	0.030	1.889	0.169	1.04 (0.98–1.10)
DRG weight	−0.075	0.176	0.181	0.670	0.93 (0.66–1.31)
Charlson score	0.112	0.077	2.434	0.119	1.12 (9.72–1.29)
Referred to TOC	0.751	0.410	3.356	**0.067**	2.12 (0.95–4.73)
Attended TOC	−1.684	0.804	4.383	**0.036**	0.19 (0.04–0.90)
Principal diagnosis					
Septicemia NOS (038.9)	0.376	0.498	0.571	0.450	1.46 (0.55–3.86)
Cerebral artery occlusion NOS with infarction (434.91)	−0.368	1.024	0.129	0.719	0.69 (0.09–5.14)
Pneumonia, organism NOS (486)	.408	0.740	0.304	0.581	1.50 (0.35–6.41)
Obstructive chronic bronchitis with acute exacerbation (491.21)	1.294	0.617	4.395	**0.036**	3.65 (1.09–12.23)
Acute and chronic respiratory failure (518.84)	0.149	1.024	0.021	0.884	1.16 (0.16–8.63)
Acute kidney failure NOS (584.9)	0.305	1.024	0.089	0.765	1.36 (0.18–10.09)
Subendocardial infarct, initial (410.71)	−11.841	440.814	0.001	0.979	0
DM type 1 with ketoacidosis, uncontrolled (250.13)	2.345	0.499	22.038	<**0.001**	10.43 (3.92–27.77)
Urinary tract infection, NOS (599.0)	−11.841	471.250	0.001	0.980	0
*E. coli* septicemia (038.42)	0.564	1.024	0.303	0.582	1.76 (0.24–13.06)
